# Standpoints of Roma women regarding reproductive health

**DOI:** 10.1186/s12905-015-0195-0

**Published:** 2015-04-28

**Authors:** Marjeta Logar, Danica Rotar Pavlič, Alem Maksuti

**Affiliations:** Clinical Department of Maxillofacial and Oral Surgery, University Medical Centre Ljubljana, Zaloška cesta 002, Ljubljana, Slovenia; Faculty of Medicine, University of Ljubljana, Poljanski nasip 58, Ljubljana, Slovenia; Faculty of Information studies, Novo mesto, Sevno 13, pp.299, Novo mesto, Slovenia

## Abstract

**Background:**

Little is known about the reproductive health of Roma women in Slovenia. The aim of this study is to present the standpoints of Roma women regarding reproductive health, the degree to which primary healthcare services are available to Roma women and the reproductive health circumstances which lead most Roma women to decide to visit a gynaecologist.

**Methods:**

A qualitative research study was carried out. Forty-four adult Roma women from the Hudeje/Vejar settlement in the Dolenjska region, Slovenia, took part in the research. The collected material was processed by means of inductive (qualitative) content analysis. The coding procedure was supported by the QDA software Atlas.ti.

**Results:**

Eighteen categories and six themes were identified that enable with the relevant codes an understanding of the standpoints of Roma women regarding reproductive health. The research results showed that the cultural needs of Roma women should be taken into account in their comprehensive healthcare treatment. Roma women wish for equal treatment when health is in question, drawing attention to better communication and the problem of ethnic discrimination in medical facilities. Roma women also feel a need to be educated and to receive professional advice, such as appropriate lectures and/or workshops dealing with reproductive health that would ensure them a higher quality of life over time.

**Conclusions:**

The research results call attention to the necessity of recognising both the need to educate Roma women as well as the need for different approaches to the provision of healthcare services in the field of reproductive health with such a sensitive group of female inhabitants. It will be necessary to familiarise them with preventive programmes and to implement such programmes, to inform them of possible diseases and to encourage them in a friendly and easy-to-understand manner to regularly visit their gynaecologist.

## Background

Reproductive health entails the condition of complete physical, mental and social well-being in all reproductive functions and processes, not only the absence of disease and weakness. It includes the right to a satisfying and safe sexual life, free decision-making regarding having children, equal accessibility of the knowledge concerning family planning and the right to medical services which ensure women a safe pregnancy and labour as well as effective prevention and early detection of illnesses [1].

Individual ethnic groups, such as the Roma people, have their own specific health cultures, which change under the influence of other cultures [2]; however, especially in regard to care for reproductive health, the traditions and connectedness with the cultural and historical starting points of the Roma community, with respect to the values and functioning in the group, are still present [3]. Some studies have shown that Roma women are burdened because of their family position, lower education, unemployment, poverty and high degree of social risk [4]; furthermore, communication with medical staff in healthcare institutions is an obstacle [5].

Scientific research studies regarding the reproductive health of Roma women indicate that different obstacles can be encountered in basically all of Central and Eastern Europe (CEE). There are some structural and systemic constraints: the lack of financial means is increasing, difficulties in obtaining health insurance are increasingly greater [6] and dependence on formal and informal forms of payment for healthcare services is increasing [7]. In some countries, the scope of healthcare services already restricts family planning, and prenatal care is not included in insurance schemes; consequently, contraceptive use is low [8] or there is reliance on more traditional and unsafe methods such as withdrawal and the lactational amenorrhea method [9,10]. Moreover, early marriages are prevalent, the adolescent birth rate is high [11] and bad conditions lead to a risk of complications during the pregnancy [12]. The substandard healthcare infrastructure in the countryside and a lack of healthcare providers in the Roma settlements of large cities are an obstacle [13]. Roma women are also faced with racial discrimination [14]. Roma gender inequality also exists as a broader cultural pattern in all areas of life [15]. Roma people are only barely informed or aware of reproductive health issues [16], which could be connected to the poor school enrolment of the Roma population in almost the entire CEE region [11,17-19].

In Slovenia, Roma women encounter various difficulties in taking care of their reproductive health; however, these difficulties are not as a whole comparable to the obstacles in other countries. Reproductive health laws for the Roma population in Slovenia are not fully regulated; there is no unique birth control model (e.g. reproductive or parental strategy [20]) and there is a lack of data about induced abortion and data surveys about the health status of the Roma population compared to some other countries in the CEE [14,9-11,19]. Roma people in Slovenia have the status of an equal ethnic minority group with full rights and even some elements of positive discrimination. In the Republic of Slovenia, the position of the Roma people is comprehensively regulated by the Act on the Roma Community in the Republic of Slovenia, which also ensures the exercise of special rights in the field of healthcare [21-23]. Thus, formally speaking, Roma women in Slovenia have access to the public healthcare system equal to that of other inhabitants [24], but in practice they often do not utilise these possibilities sufficiently. There have been several actions to increase “health literacy” and empowerment as regards healthcare services among Roma women [25]; namely, not long ago Roma women in Slovenia sought medical aid or healthcare services only in the event of an injury [26]. It is encouraging that in 2009 the 2^nd^ National Conference on the Health of Roma People was organised, and the conference was focused on the health of Roma women. The conclusions of the conference stressed that Roma women should be better informed about the role of reproductive health, that the importance of preventative gynaecological examinations should be emphasized to them and that approaches appropriate to their culture and programmes promoting health should be developed [27].

In spite of the possibilities and progress, it has been stated that in Slovenia, differences in health between members of different social groups exist [28]. The reasons can be found in groups’ different health treatment due to different personal factors, such as ethnicity, a physical or linguistic inaccessibility of healthcare services (e.g. a lack of understanding of the Slovene language), psychological inaccessibility (e.g. distrust) and others [29]. Inequality regarding healthcare exists between the regions and amongst the Roma populations [30]. Thus, numerous Roma people in the area of the Dolenjska region, which is where the Roma settlement of Hudeje/Vejar is situated, live in residential conditions that have improved only in the last decade [31]. Progress in regard to the improvement of social circumstances, education and the accessibility of healthcare is noticeable. However, weaknesses can be observed in poor health awareness and unhealthy lifestyles, a lack of participation in preventive programmes, the inappropriate use of healthcare services and the inappropriate approaches to providing healthcare services to Roma women [29]. Healthcare workers see developmental opportunities in helping the Roma people be more aware of the possibilities offered by healthcare services and in including the Roma people in health education programmes adapted to the Roma people, both in terms of their content and regarding their temporal and spatial accessibility [32].

In Slovenia, no research has previously been carried out that has evaluated the standpoints of Roma women regarding reproductive health. In our research, we examined the degree to which Roma women still stick to traditional approaches regarding reproductive healthcare, whether they are acquainted with their healthcare possibilities and the degree to which they make use of the available opportunities to maintain reproductive health in the existing healthcare system. The greatest scientific contribution of our research is in presenting cases and the real-life experiences encountered by Roma women in realising their needs with regard to reproductive health.

## Methods

### Fieldwork and data collection instrument

The fieldwork ran from December 2013 until September 2014 and included discussions with a representative from the Roma community, a social worker, the Director of the Trebnje Centre for Social Work and the employees of the Trebnje Health Care Centre^a^. In this way, it was possible to approach individual Roma women in order to carry out the interviews with a culturally sensitive attitude. The interviews were held between 16 May 2014 and 6 August 2014^b^. The sample size was not fixed prior to data collection; this did not depend so much on the resources and time available but more on the number of Roma women prepared to participate in our study. A purposive sample size was determined on the basis of the theoretical saturation, which is the point in the data collection process when new data no longer bring additional insights to the research question [33]. According to this condition, the final number of interviews conducted was 44.

We used interviews as data collection tool. In accordance with the research goals and specificities of the target population, we decided to conduct so-called focused interviews [34,35], for which it is characteristic that the theme of the discussion is known in advance and that data acquisition and data interpretation are carried out in an open manner. The starting point for the discussions was a semi-structured questionnaire prepared in advance. In addition to demographic variables, it also contained questions on the perceptions of Roma women regarding their reproductive health, the factors influencing their use of healthcare services at gynaecological clinics and the role of healthcare services in the field of reproductive health. Within the framework of these questions, the interviewed women could respond to all questions, sub-questions and encouragements. Due to the cultural and other specificities of the researched population, for the individual themes we prepared – in addition to the focused questions – pictorial material, posters from workshops and notes from various events.

### Demographic characteristics of participants in the study

Forty-four adult Roma women from the Roma settlement of Hudeje/Vejar in the municipality of Trebnje in the Dolenjska region of Slovenia were included in the study. As the most common sampling strategy in qualitative research [33], we used a purposive sample of participants relevant to our research question. The socio-demographic characteristics of the interviewed women are presented in Table 1. Only Roma women who voluntarily chose to speak about their feelings and views regarding reproductive health took part in the study. The Roma women were participatory subjects of the research. We focused our attention on only one local community since in Slovenia there are great differences between Roma communities as to way of life, consideration of tradition, the degree of socialisation and their inclusion in the environment [2].

**Table 1:** **Socio-demographic characteristics of the interviewed Roma women by age group, status, education and health insurance**

		**Marital status**			**Education**				**Health insurance**	
**Years**	**Number**	**Married**	**Single**	**Widow**	**Illiterate**	**Incomplete primary school**	**Completed primary school.**	**Completed secondary school**	**Yes**	**No**
18-24	8	1	7	-	1	4	3	-	6	2
25-30	8	5	3	-	-	4	4	-	8	-
31-40	7	6	1	-	-	5	2	-	7	-
41-50	7	4	3	-	1	4	1	1	7	-
51-60	8	4	4	-	4	4	-	-	8	-
61 and more	3	2	-	1	3	-	-	-	3	-
No data	3	2	1	-	1	2	-	-	3	-
**Total**	**44**	**24**	**19**	**1**	**10**	**23**	**10**	**1**	**42**	**2**

### Method of data analysis

We conducted a qualitative study using the method of qualitative content analysis. The key characteristic of this method is that extensive texts are classified into smaller content categories. The method contains an initial phase of preparation and organisation, including open coding, category formation and abstraction [36-38]. As a tool to facilitate qualitative data analysis, we used the software *Atlas.ti*. We employed the software in an open coding procedure based on breaking data apart and delineating concepts to represent blocks of raw data and qualified those concepts (coding units, categories and themes) in terms of their properties and dimensions [39] based on a theoretical framework of reproductive health.

After determining the coding units, we used open coding for the identification of categories and their classification [36]. First, we carried out a thematic analysis of the content. We started with the preparation phase, which included the selection of analysis units. In our notes and transcripts of the interviews, we marked those parts of the texts that we needed for further elaboration and that were connected with the research goals – that is, a sentence or more expressing a relevant declaration. In the qualitative synthesis, we used codes and categories, which means that we classified the units with respect to their meaning [37].

Written consent was obtained from all participations of in the researsch.

## Results

On the basis of the obtained results, the three researchers, who independently coded the transcripts of the interviews with the Roma women, formed 18 categories in which 82 codes with a total frequency of 457 were identified. From the categories, we formed six themes referring to the reproductive health of the Roma women: 1) Roma women’s personal health; 2) dealing with reproductive health in the Roma community; 3) treatment at the gynaecologist; 4) the influence of circumstances on the accessibility of healthcare services; 5) (desirable) comprehensive treatment and 6) educational programmes on reproductive health for Roma women. Table 2 presents the themes and categories with the total number of identified codes.

**Table 2:** **Survey of the fields, categories and themes regarding the reproductive health of Roma women in the Hudeje/Vejar settlement**

**Categories**	**Number of codes in the individual category**	**Themes**
- personal healthcare	85	- Roma women’s personal health
- fears regarding health as a cultural specificity	21	
- reproductive health and the role of the family	35	- dealing with reproductive health in the Roma community
- beliefs and self-medication	25	
- circumstances of a visit to a gynaecologist	9	- treatment at a gynaecologist
- regular consultations with a gynaecologist	52	
- temporary visits to a gynaecologist	7	
- understanding instructions at the gynaecological clinic	15	
**-** attitude toward the gynaecologist	14	
- social-economic problems as an obstacle to treatment	10	- the influence of circumstances in society on the accessibility of healthcare services
- rights, laws	23	
- attitudes of Roma women toward healthcare services, communication	74	- (desirable) comprehensive treatment
- occurrence of stigmatising or discrimination at the clinic	25	
- inclusion in the network of preventive examinations	8	
- participation in educational events	11	- educational programmes on reproductive health for Roma women
- reasons for non-participation	12	
- the quality of educational programmes	21	
- educational strategies for Roma women regarding the reproductive health of Roma women	10	

### Roma women’s personal health

The results showed that for Roma women, health is highly valued. One of the interviewed women summarised this in the following statement: *“Everything will be fine, as long as one is healthy” (R10).* They expressed an interest in personal healthcare, such as in their attitude to smoking, which would be given up by most women during pregnancy, but by some of them even not in this period: *“Yes, of course I would, but some do no stop smoking. Yes, I would stop” (R4).* They expressed the importance of diverse nutrition, regular meals and maintaining one’s body mass: *“One plate of salad is not enough. Potatoes should also be included” (R17).* Their attitude to health can be summarised by the declaration: *“If possible, you must rely on yourself” (R34).*

Fear as related to lack of health is very noted by Roma women, especially fear of diseases and their consequences, such as cancer: *“Will the doctor say something good or bad? He should not say something bad straight to my face ....he just scares me” (R8).* Roma women are especially worried about their children: *“I am afraid that something would not be correct with the child” (R13).* They often cannot distinguish dangerous conditions from ones that are not dangerous, and they do not understand the reasons for the disease; they experience disease exceptionally emotionally. They find support in the family, which usually accompanies women to the physician. One interviewed Roma woman explained it thusly: *“You know, what the Roma people’s habit is, when one of them comes, then the others also come. If some extreme situations occur, if somebody collapses, many of them come” (R17).*

### Dealing with reproductive health in the Roma community

In the Roma community, the family has a special role also in connection with questions of reproductive health. The interviewed Roma women first try to find answers concerning reproductive health difficulties, menstruation, pregnancy, contraception and birth from their mothers. In the interviews, they often stated: *“My mother was the one who primarily explained this to me” (R33).* Even just two decades ago, some mothers still helped their daughters in labour. When asked where they had given birth, some Roma women said that it had happened at home, with the assistance of their mother or relatives who had some knowledge of this: *“We didn’t go to the physician; the first time I gave birth in the hospital, then twice simply at home, at my mother’s. She was like a midwife. She also had helped herself alone” (R15).* Some of them said that Roma women still went to relatives for advice, but to a lesser degree: *“They first tell you about their experiences, then direct you to the physician” (R4).* This information about pregnancy remains a matter of family. They say that with regard to a pregnancy, they first inform their *“husband and mother, nobody else. Such is the custom of the Roma people, girls don’t say anything about it for even five months, and by then it can be already seen” (R17).*

Developments in society have contributed to the changing attitudes of Roma women to reproductive health. In the interviews they said that in addition to their mother as their first confidant and adviser, nowadays they also go to physicians: *“Mothers, but also in the clinic they tell us. Well, now, the young women mostly listen to physicians” (R10).* Most answers can be summarised with this statement: *“Well, I prefer to discuss these things with the physician; he advises me the best” (R1).*

In discussions on gynaecological health and making decisions, such as about contraception, husband and wife are frequently not considered equal. One of our interlocutors said: *“he* (the husband) *said that ‘we will do what I decide. That it is my decision’” (R33).* Although she believes that the decision is hers, she is trapped in the loop of gender inequality and male dominance, which is consistent with Roma tradition and the above-mentioned cultural patterns. This is the same in the case where attention is drawn to distrust between the male and female, where the latter shows a certain degree of discomfort when there is talk about reproductive health (e.g. contraception and protection against sexually transmitted diseases) in the presence of men (see below).

Self-medication and resorting to various beliefs, which in the past were the only known customs, are barely observable in Roma communities these days. The women explained that in event of pain, they take a pill: *“I believe that it helps more (R42);* sometimes they simply help themselves: *“When we have strong menstruation, we take a cold shower on the lower part of the belly, and then it flows less” (R30).* Older people still use medicinal herbs: *“Lime-blossom tea, marjoram, caraway. Lime-blossom is for the heart, caraway for the stomach. We bite or cook it. It is also for children, if something is hurting them. Marjoram – if you have a headache or a sore throat” (R44).* Roma women often request help from God if they have health problems: *“I’ll go to Brezje* (a pilgrimage); *Mary will help. I have been to Brezje three times already” (R 19).*

### Treatment at the gynaecologist

Roma women visit the gynaecologist primarily when they have difficulties connected with pain or when they run out of medicine. They define their need to visit the gynaecological clinic thusly: “*When something is wrong” (R3);* “*When I feel ill” (R10); “When bleeding occurs, someone goes immediately to the gynaecologist” (R31)* and *“If I am pregnant” (R25).* When they are pregnant, young Roma women regularly come to the clinic for examinations; after delivery, they mostly take care of contraception. Their attention to reproductive health ends after some time, and they return for treatment only in the event of problems, such as: *“When I was pregnant, I went to the doctor and asked for contraception. We don’t need more children, really. In the past women didn’t know this and had even 11 children” (R42).* They stated that they would come to an examination upon invitation, but such invitations are rare.

The Roma women understand the instructions that they are given in the gynaecological clinic: *“Yes, in Slovene, we are not such stupid girls that we wouldn’t understand” (R29).* If necessary, they also obtain additional explanations. They state: *“They explain” (R14). “If not, I ask once again and she explains” (R20). “They also show a picture” (R25).* They say that it would be better if gynaecologists gave information in a more understandable way and in simple words. The women we interviewed also expressed their opinions about their different feelings when visiting a general practitioner or a gynaecologist. They answered: *“Yes, this is more personal, this examination” (R35). “A little, no, uneasy, it’s not the same when you go to a real physician. One must get used to it” (R28).*

### The influence of circumstances on the accessibility of healthcare services

Of the interviewed Roma women in the Hudeje/Vejar settlement, two of them who had come from another country and/or some other area of Slovenia had no health insurance. Roma women are well informed about managing health insurance as well as the rights deriving there from. In their explanations, they were very precise: *“Yes, we have social assistance benefits, we have health insurance. If we were to lose social assistance benefits, then we no longer would* (have health insurance)*” (R22).*

More serious obstacles regarding access to healthcare are represented by some other socioeconomic problems, such as poverty. The common practice in healthcare centres is to make an appointment by phone. However, many Roma people cannot afford phones: *“Where will I get a €5 phone card? That is more than 1 kg of bread for the children. I was angry. Sometimes I even sit and wait for three hours” (R11).* The interviewees explained that older women depend on their relatives or other inhabitants from the settlement for transportation to the healthcare centre, and that this transportation needs to be paid for. As a rule, Roma people take only relatives into their cars; they prefer to avoid others. Difficulties are especially apparent regarding growing one’s own vegetables. Roma people have small gardens, but, as a Roma woman stated: *“We have small gardens, but there are also many rats and mice … I’ll poison myself, I am afraid to eat such vegetables” (R9).* The bad hygienic circumstances also include a lack of running water or sanitary facilities and bathrooms. The Roma women wish to improve healthcare by reaching a hygienic minimum with their financial participation and, consequently, to take care of their health. One of them proposed: *“At least a WC, so that girls would have one … I gave €500 of my own money for a sewage system … if I had known, I wouldn’t have given anything” (R9). “My little girl says that she would like to wash herself. How can she? I say to her: Wash yourself in the washbasin. Well, I would like to help, and the municipality should help, but it hasn’t” (R9).*

As an obstacle concerning access to the maternity hospital, one of the interviewed women stated: *“As my other children were already bigger and I could go to the hospital … they could look after the smaller ones at home .... before I didn’t dare to leave them at home” (R43).*

### (Desirable) comprehensive treatment

As affirmed by most of the interviewed women, communication with healthcare professionals is tolerable and carried out with understanding, additional explanations and clarification of instructions: *“They are good, friendly, they explain everything, they do not shout, they call us immediately to the doctor” (R22). “If I have a pain, they give medicine. They help. They behave nicely – to everyone” (R7).* However, within the answers were also divided opinions regarding the adequacy of communication with Roma women, such as: “*We get along with some of them, but some of them we don’t get along with” (R29).* Then the Roma women feel inferior, discriminated against and misunderstood: *“The other day I came and was waiting. A young nurse was there and she said, ‘And what is this all about?’ I said – madam, don’t be angry, that’s why you are here. If we had a school of our own, my daughter could also do such a job” (R11).*

Roma women are encouraged to have preventative examinations, but they rarely choose to subject themselves to such examinations: *“I’ve received the invitation more than 15 times and I haven’t gone” (R39).* Most of them need additional encouragement: *“Well, the gynaecologist says I should go. So I go” (R8).* This should be understood as the appeal of or the desire for more comprehensive treatment based on improved communication and deeper involvement by medical staff understood merely as the main non-Roma authority. Deeper explanation could be sought in the idiosyncrasies of Roma culture and their specific relationships with non-Roma (gadje), which is especially renowned in the field of health [2].

### Educational programmes on reproductive health for Roma women

The Roma women were offered education at both the healthcare centre and in the settlement. Some of them had attended lectures. They mostly evaluated them positively: *“Once a nurse came from Trebnje. Only women were present. It was interesting and necessary, as some of the women previously did not know the information”* [4]*.* In any case, they do not want themes about “*ugly diseases” (R1),* rather themes more “*about contraception, pregnancy, delivery, care for the child” (R25), “breastfeeding” (R29)* and *“about the health of women” (R37).* The organisation of educational programmes in the healthcare centre is problematic – namely, according to one of the participants, the problem concerns the organisation of time: *“They are not all consistent, there is no sense, some simply forget” (R5).* They found the discussion on the need to organise education for young Roma women within the settlement stimulating, including the idea that it be only for women: *“Yes, men must not be present. I feel so ashamed of myself. Only for women” (R17).* When asked if some of them would attend workshops, they answered: *“Yes, of course I would go. With pleasure” (R38).* The Roma women explained that educational programmes should be organised in their settlement in the form of workshops, presentations and discussions: *“So you could come several times, ask girls how they are … there are so many things that are harmful, but we don’t know about them” (R10).*

## Discussion

According to quality criteria in qualitative research [40], we can say that our study meets all the necessary criteria of unbiased and methodologically proper qualitative research. The data collection procedure was done in accordance with the tenants of focused interviewing, which is characterised by openness, trust, willingness to collaborate and the lowest possible power-difference between researchers and informants. As an extremely important and widely used method for qualitative data analysis [36,38], content analysis was used to describe the content regarding the perceptions of Roma women regarding reproductive health. The research offers new information about the extent to which Roma women still maintain traditional approaches to preserving reproductive health, whether they are familiar with the various ways of ensuring health and their use of the services provided by the healthcare system to ensure reproductive health. The greatest contribution of the research lies in the fact that it reveals cases and real-life personal experience of Roma women regarding reproductive health. However, at this point we should also point to several limitations of our study.

The first general limitation is related to epistemological criteria and validity in qualitative research [41]. Although they provide richness in detail, large-scale representative quantitative surveys are needed to capture a large amount of data and shed more light on the reproductive health needs of the Roma population in Slovenia. A second limitation is linked to the purposive sample used in the study. We had to focus our analysis on just one Roma settlement in one Slovenian region, which is further linked to the limited number of the participants in the case study. Through a non-probability sampling technique, the participants in our study were selected according to subjective judgments and the willingness of the Roma women to participate. Although the subjective aspect of purposive sampling is a major disadvantage only in qualitative research [42], we are aware that a study with random participant selection and a quantitative approach would ideally complement our small-scale qualitative study. Finally, only Roma women as healthcare users participated in our study. For a more comprehensive picture, healthcare providers (doctors and other healthcare workers) and policy makers should also be included in research to provide additional insight. However, these limitations could also be understood as a starting point for further research in the field of ethnic minorities and their healthcare needs.

The participants in our research were aware that in order to preserve health, it is necessary to act on several levels, first of all with regard to personal healthcare. We believe that the unhealthy lifestyles, lack of nutrition and physical inactivity of the interviewed women are not representative of Roma culture, with the exception, perhaps, of an exaggerated dependence on smoking [43]. A lack of money, poor hygienic conditions and a general poor level of information prevent the Roma women from ensuring their healthcare in the manner and degree to which it is realised by the Slovenian majority population [15]. This confirms the findings of studies from other CEE countries, for example those from Serbia, where socio-economic problems, poor school enrolment and the maintenance of traditional patterns by local Roma affect their reproductive health [9,10,20].

Regarding the Roma women who cooperated in our research, we conclude that the family is an important factor regarding trust and seeking a cure for an illness. During pregnancy and around the time of delivery, the younger generation (18 to 30 years old) visit the gynaecological clinic more frequently; and as a general rule, they give birth in the maternity hospital. This could be the result of recent initiatives (e.g. lectures and workshops in Roma settlements) related to increasing “health literacy” and empowerment in regard to healthcare services among Roma women [25] and the willingness of the younger population to attend related events. We stated that a Roma woman decides on contraception together with her partner and that the younger people (18 to 30 years old) carefully plan their families. However, the results from our study are not sufficient to ensure that traditional (reproductive) parental care [20] is no longer transmitted to new generations. Since this was not the aim of our study, in the future, additional research is needed using different methods and at the population level.

The research results are not in accordance with the general opinion that in pursuit of their reproductive health Roma women do not visit gynaecological clinics. They are afraid of such contact due to a lack of knowledge about health because. They have not had many positive experiences with healthcare workers because of their personal views regarding gynaecological examinations (e.g. different feelings when visiting a gynaecologist from those experienced when visiting a general practitioner) such as shame, discomfort or distrust, which are embedded in their cultural patterns [2,44]) and also due to difficulties with child care and the new way of arranging an examination, namely by phone. A call from a mobile phone is a great expense for Roma women, and we can see the results in how they then organise their time. With no phone access, they have to sit in the waiting room for hours, which is unacceptable for them and discriminatory from the side of the majority population. In healthcare centres, the possibility for “walk-in” treatment should be implemented, as has been introduced in other countries [45,46] because the current system of making medical appointments has failed with regard to some people. Roma women thus come to the clinic in groups or they go to the emergency room, which makes a comprehensive approach to the treatment of patients and pregnant women impossible.

Regarding the reproductive health of Roma women, we must not only deal with their attitudes toward healthcare but also with the accessibility of healthcare services. This is connected with the public system of health insurance in Slovenia, which, under certain conditions, enables all services to be covered by compulsory health insurance. In this context, Slovenian Roma people undoubtedly have better conditions than, for example, Roma people in Bulgaria, who have to bring their own consumables in medical facilities if they want to receive delivery care, although approximately two-thirds of them do not have the financial resources for the necessary medications [6,19]. Another example is Macedonia, where Roma mothers often cannot afford regular antenatal care visits or other health services that are formally free and subsidised by vertical prevention programmes. In Slovenia, where free access to healthcare services for Roma women is enacted by law [21], we can state that Roma women rarely co-operate in preventive programmes, and after the birth of their children, they visit a gynaecologist less frequently. We ascribe this fact primarily to a lack of understanding of the Roma culture and the approaches to the Roma population by healthcare services, although the staff members of Trebnje Healthcare Centre have established good communication with the local Roma population.

The findings regarding communication with healthcare professionals cannot be generalised although it has been proven that in Slovenia the existing knowledge of healthcare workers does not provide sufficient support to ensure an appropriate approach to Roma women with regard to their cultural specificities [47]. If we compare this with situation in other CEE countries, we can recognise a general trend of intolerance. Evidence of discrimination against Roma when accessing health services can be found in the majority of the referenced studies [4-8,13,14,16,19]. In some cases, especially at the time of pregnancy and childbirth, Roma women are even more susceptible to abuse and discrimination by health professionals [6]. All of this calls for a careful consideration not just inside the research framework of minorities’ health rights but also in the context of the broader problem of ethnic discrimination and intolerance in our society.

Our modest contribution suggests that in order to increase the quality of Roma women’s reproductive health, successful communication with healthcare workers is necessary. Thus, in addition to professional knowledge, the special engagement of healthcare services in the Trebnje gynaecological clinic will be required to ensure a better response to the needs of Roma women concerning maintaining reproductive health. It will be necessary to employ assertive behavioural skills and intercultural dialogue by using simpler words, ensuring mutual respect and being willing to openly discuss matters. This should be based on trust and mutual respect. Only in such a manner can good relations with Roma women be established, which will improve their lifestyle, health and recognition of being accepted in society.

The efforts of Slovene society to ensure community-based concern for the reproductive healthcare of Roma women at the primary level is not a novelty – recently, such efforts have even been increasing – however, in this period of economic crisis, there is the risk that free health insurance may be cancelled and that the process of improving the residential and social conditions and education and employment of Roma people may come to a halt [32]. The interviewed Roma women in the Hudeje/Vejar settlement emphasised the exceptional importance of education and information about their rights, their changed lifestyle, the importance of health for the whole family and their own reproductive health. The latter is important in the context of greater care for the reproductive health of this group, as educated Roma women like to share their knowledge with the whole community. Of course, understanding and tolerance by the majority population, public policy creators and policy decision-makers are also welcomed.

## Conclusions

In this study, we describe the circumstances in which Roma women from the Hudeje/Vejar settlement in the Dolenjska region decide to visit the gynaecological clinic and to what degree primary reproductive healthcare services are accessible to them. The study therefore indicates that comprehensive treatment of Roma women regarding reproductive healthcare should be based on balanced efforts towards quality treatment in healthcare centres, with due consideration of the cultural specificities of Roma women. Community-based healthcare for Roma women does not entail only the efforts of the healthcare services, but also the promotion of Roma women’s own responsibility for their reproductive health. Our research findings also emphasise that care for the reproductive health of Roma women can still be improved with planned scientific research and consideration of these findings in practice.

Future work should therefore include follow-up on the progress in the field of Roma women’s reproductive health. Comprehensive research frameworks should be used to investigate this subject further and to include all involved stakeholders. In the future, it would be reasonable to conduct research on the level of the entire country, which would include all Slovenian regions where Roma people live. Such a national research study would enable a comparison between individual regions regarding the attitudes of Roma women to reproductive health and represent a conceptual framework for research on these problems in other countries with Roma populations or other ethnic minorities.

## Endnotes

^a^The study was approved by the National Commission for Medical Ethics (No. 23/4/14, date: 8 April 2014). The document in written form is available from the authors.

^b^Marjeta Logar and Nataša Krese carried out the interviews.
